# Fracture behaviors of ceramic tissue scaffolds for load bearing applications

**DOI:** 10.1038/srep28816

**Published:** 2016-07-12

**Authors:** Ali Entezari, Seyed-Iman Roohani-Esfahani, Zhongpu Zhang, Hala Zreiqat, Colin R. Dunstan, Qing Li

**Affiliations:** 1 School of Aerospace, Mechanical and Mechatronic Engineering, The University of Sydney, Sydney, NSW 2006, Australia

## Abstract

Healing large bone defects, especially in weight-bearing locations, remains a challenge using available synthetic ceramic scaffolds. Manufactured as a scaffold using 3D printing technology, Sr-HT-Gahnite at high porosity (66%) had demonstrated significantly improved compressive strength (53 ± 9 MPa) and toughness. Nevertheless, the main concern of ceramic scaffolds in general remains to be their inherent brittleness and low fracture strength in load bearing applications. Therefore, it is crucial to establish a robust numerical framework for predicting fracture strengths of such scaffolds. Since crack initiation and propagation plays a critical role on the fracture strength of ceramic structures, we employed extended finite element method (XFEM) to predict fracture behaviors of Sr-HT-Gahnite scaffolds. The correlation between experimental and numerical results proved the superiority of XFEM for quantifying fracture strength of scaffolds over conventional FEM. In addition to computer aided design (CAD) based modeling analyses, XFEM was conducted on micro-computed tomography (μCT) based models for fabricated scaffolds, which took into account the geometric variations induced by the fabrication process. Fracture strengths and crack paths predicted by the μCT-based XFEM analyses correlated well with relevant experimental results. The study provided an effective means for the prediction of fracture strength of porous ceramic structures, thereby facilitating design optimization of scaffolds.

Current therapeutic strategies for bone defects include autografts, allografts and other synthetic substitutes such as metals and ceramics^1^, which all have their own problems and limitations^2^. In particular, the existing synthetic bone substitutes available in clinic are of inadequate mechanical strength, limiting their extensive applications and leading to suboptimal outcomes including non-unions and fractures. The mechanical competence of a synthetic biomaterial is critical for load-bearing applications. Currently available 3D ceramic scaffolds, including hydroxyapatite (HAp), β-tricalcium phosphate (β-TCP), bioactive glasses^3^,^4^, and more recently calcium silicates^5^,^6^, are considered to be brittle^7^ and fracture easily, making them unsuitable for bone regeneration in load-bearing scenarios^8^,^9^.

While substantial experimental studies have been conducted to evaluate the fracture strength of tissue scaffolds^10^,^11^,^12^, to the authors’ knowledge, little work has been reported in the literature concerning the computational modeling and analysis of fracture behaviors of ceramic scaffolds with the exception for some typical stress analyses. For example, Miranda *et al*.^13^,^14^ applied conventional FEM to characterize stress fields and subsequently estimate the strength of HAp and β-TCP scaffolds fabricated by a robocasting technique. Since the structural behaviors of ceramic materials are attributed to the crack initiation and propagation prior to fracture collapse, conventional FEM may not be able to predict the fracture behaviors of ceramic structures properly.

In order to take into account the structural resistance to crack propagation, more robust computational methods capable of modeling crack initiation and propagation are crucial^15^. Numerical techniques available for such applications can basically be categorized into three main approaches, namely the discrete inter-element crack method^16^, the embedded discontinuity approach^17^,^18^, and the extended finite element method (XFEM)^19^, among which XFEM has shown superior kinematic features and numerical robustness^20^. XFEM extends the conventional FEM by enriching the solution spaces through partition of unity concept^21^, thereby enabling the problems of discontinuities and localized deformation, such as cracks, to be analyzed properly. Over the last decade or so, effectiveness of XFEM for modeling cracks and discontinuities has been validated extensively in different engineering problems^22^,^23^. However, no such study has been reported for ceramic scaffolds to date.

In the current study, XFEM was employed to model crack initiation and propagation in scaffolds made of Sr-HT-Gahnite material^10^,^24^, which possesses better strength and toughness inherently attributable to its unique microstructural features. Numerical analyses were conducted based on both post-fabrication CAD models and micro-computed tomography (μCT) based models of the fabricated Sr-HT-Gahnite scaffolds in order to consider the geometric variation induced in the fabrication process. The modeling results were validated by our in-house experimental data obtained from the compression tests performed in the current study as well as our previous study^24^. The predictions of XFEM were compared with the results obtained from conventional FEM in order to demonstrate the superiority of this new modeling technique over conventional numerical methods available in literature^13^. Furthermore, the field emission scanning electron microscopy (FE-SEM) images of specimens were obtained to validate the crack initiation and propagation paths simulated by XFEM.

## Results

**Figure 1:**
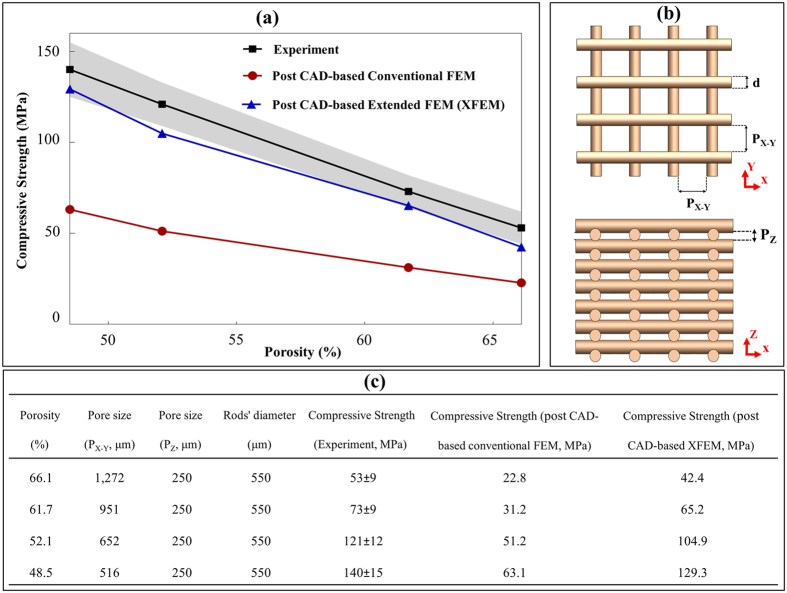
(**a**) Comparison between the previously published experimental data^24^ and numerical simulations of the scaffolds’ compressive strength (the shaded area represents the upper and lower range of the experimental data); (**b**) definition of geometric features; (**c**) details of geometric features and mechanical properties of the scaffolds.

As mentioned before, in addition to the experimental data obtained from the scaffolds fabricated in the present study, the mechanical properties of four different sets of the Sr-HT-Gahnite scaffolds, which were experimentally evaluated in our previous study^24^, were also used here to further verify the XFEM modeling results. The detailed geometric features of these four sets of scaffolds as well as their corresponding experimental data^24^ and the post CAD-based numerical analysis results are summarized in Fig. 1.

The results compared in Fig. 1 demonstrate that the post CAD-based XFEM predictions agree fairly well with the previously published experimental results, which almost lie in the lower range of the experimental data (Fig. 1). Note that the average discrepancy between the mean value of the published experimental data^24^ and the post CAD-based XFEM results is less than 13%, whereas the discrepancy between the experimental results and post CAD-based conventional FEM predictions (Fig. 1) is significantly higher (about 57%).

Furthermore, five rectangular Sr-HT-Gahnite scaffolds with an average porosity of 63.7% were prepared in this study followed by the experimental measurement of their compressive strengths. In order to investigate the effect of geometric variation induced by the fabrication process on the modeling results, the numerical analyses were conducted based on both the post CAD model and μCT models for these five scaffolds.

**Figure 2:**
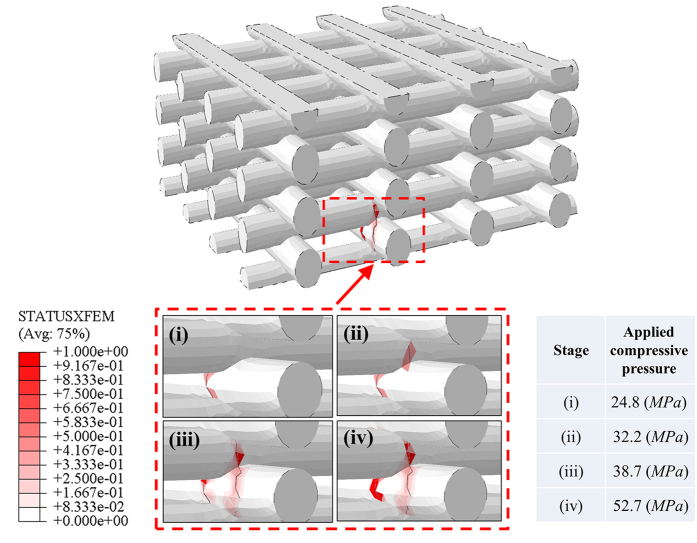
Crack initiation and propagation in a post CAD–based XFEM model related to the five scaffolds with an average porosity of 63.7% fabricated in this study.

Figure 2 illustrates the simulation of crack initiation and propagation in the XFEM analysis conducted based on the post CAD model of a typical Sr-HT-Gahnite scaffold fabricated here. We observed that when the pressure (*P*) reaches 24.8 MPa (stage (i)), the crack was initiated. It should be noted that in the XFEM analysis, stage (i) represents the initiation of crack, where some of the elements are partially cracked but the struts did not fracture completely. In contrast, in the conventional finite element analysis, this stage is considered as a complete fracture as the peak stress has exceeded the strength. As pressure *P* increases (stages (ii & iii)), the crack propagates to other regions. Once *P* reached 52.7 MPa, the two rods depicted in Fig. 2 undergo complete fracture.

**Figure 3:**
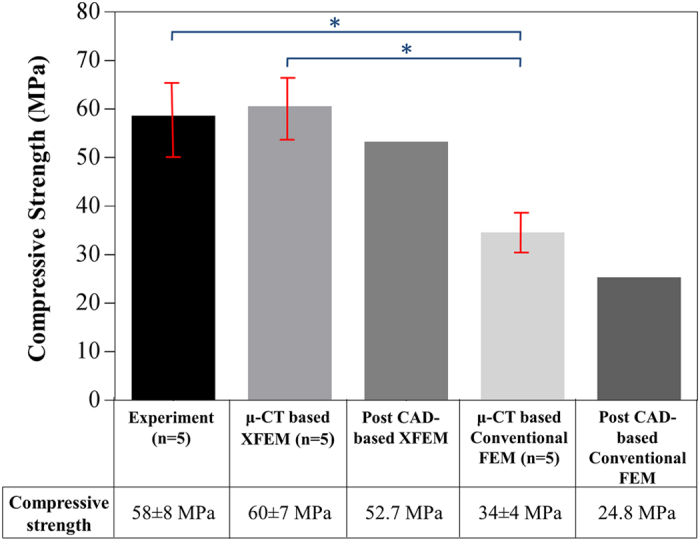
Comparison of the compressive strengths from different modeling methods conducted on the scaffolds fabricated in this study. Error bars represent the standard deviations of the predictions induced by the uncertainties in the measurement of compressive strengths, either from experimental tests or μCT-based modeling analyses. (*Significant difference between groups, p < 0.05).

The numerical analyses, including both XFEM and conventional FEM, were also conducted on the μCT models of these five scaffold specimens. The compressive strengths obtained from the μCT-based simulations were compared with the results from the corresponding post CAD-based numerical analyses and the relevant experimental tests in Fig. 3.

**Figure 4:**
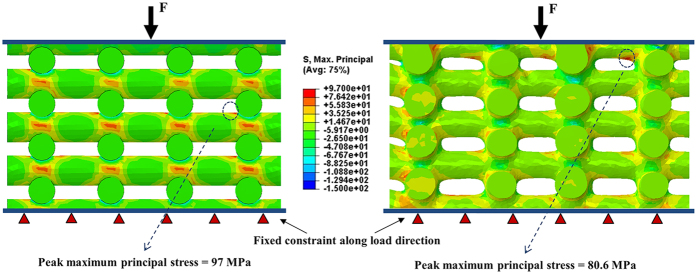
Comparison between the contours of maximum principal stress related to the post CAD-based and the μCT-based numerical models.

Not surprisingly, the comparison indicated that the μCT-based numerical analyses predicted more realistic results than the post CAD-based simulations. This is due to the fact that the μCT-based models better captured the actual specimens by modeling all details of geometric variation resulted from the fabrication process. Figure 4 compares the contours of maximum principal stress from the post CAD model and the μCT models, which further signify the fact that the stress distributions in these two models differ considerably.

**Figure 5:**
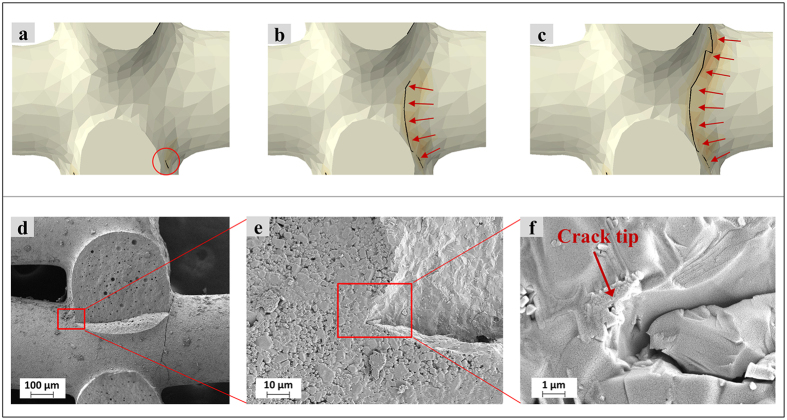
(**a**–**c**) Crack initiation and propagation path simulated by the μCT-based XFEM analysis; (**d**–**f**) FE-SEM images showing the crack initiation and fracture path related to the experimental observations.

Figure 5d–f shows the FE-SEM images related to the experimental observations, where the cracks were induced in the horizontal rods along the joints and detached the rods from the vertical rods. In order to model crack path and fracture *in silico*, XFEM analyses were conducted on the μ-CT model of the same fabricated scaffolds and the fracture path was simulated as depicted in Fig. 5a–c, which exhibited good agreement with the experimental observation.

## Discussion

This study modeled the crack initiation and propagation of the robocast Sr-HT-Gahnite scaffolds undergoing compression *in silico*. Fracture failure of ceramic scaffolds remains to be a critical issue of its kind for application in the regeneration of large bone defects under loading, mainly due to the inherent brittleness of ceramic materials. Therefore, modeling of crack initiation and propagation is paramount in order to accurately predict the fracture behaviors and mechanical strength of ceramic scaffolds. The recent development of the extended finite element method (XFEM) provides a sound methodology to undertake such a study, which enables us to model crack initiation and propagation realistically.

As shown in Figs 1 and 3, the results of the numerical simulations conducted on both the post CAD models and the μCT models confirmed that XFEM predicted the fracture strengths of scaffolds more accurately than the conventional FEM. The main reason is that the XFEM considered the material’s fracture toughness and subsequently its resistance to crack propagation during loading, which as an important factor, is neglected in the conventional FEM. In other words, conventional FEM presumes that fracture occurs when the maximum principal stress in the structure reaches or exceeds the inert fracture strength of its constituent material^25^; whereas in XFEM, this stage is assumed only as onset of crack, after which subsequent growth of the crack would be modeled in a time-dependent course according to the energy release rate of the material^26^.

As discussed earlier, the energy release rate of a material, which is directly proportional to its fracture toughness, can be a driving force for crack propagation inside sophisticated scaffold structure. Therefore, materials with different fracture toughness exhibit different resistances to crack propagation, consequently leading to different fracture behaviors and strengths in the structural systems, a fact that has been previously perceived through a parametric study^27^. The XFEM technique was applied to investigate the effect of fracture toughness on fracture strength of 2D ceramic models containing pre-defined discontinuities. It was demonstrated that an increase in fracture toughness of the material would lead to a higher fracture strength of the structure^27^. Therefore, it is crucial to take into account the fracture toughness of material in numerical simulations in order to model crack propagation and the consequent fracture properly, which has become possible by adopting the XFEM technique.

In order to take into account the actual geometry of fabricated scaffolds, numerical simulations were performed based on the μCT models of fabricated scaffolds as well. The results depicted in Fig. 3 indicate that the *in-silico* analyses conducted on the μCT models of scaffolds lead to more realistic predictions of their fracture strengths. The comparison of the post CAD-based with the μCT-based modeling results (Fig. 3) revealed that the μCT-based simulations predicted higher compressive strengths which are closer to the experimental data. The reason for this could be the difference in the distributions of stress in these two models. Indeed, sharp edges and corners, which are often the source of stress concentration in the post CAD-based models, differ from those of the μCT-based models. This can be clearly observed in Fig. 4, with a typical scaffold under the same pressure, the peak first principal stress is 17% less in the μCT-based model compared with the post CAD-based model. This justifies why the μCT-based models predicted a higher compressive strength of the scaffold structure. This also explains why the post CAD-based XFEM results (Fig. 1) lie in the lower range of the data obtained from the experimental tests.

Figure 5a–c depicts crack initiation and propagation path simulated by XFEM conducted on the μCT model of the fabricated scaffold. As shown in Fig. 5a, the crack initiated from the region marked with a red circle and then grew upward along the red arrows marked in Fig. 5b,c. It can be seen that the crack origin corresponds to the location of maximum principal stress in the scaffold seen from Fig. 4. These *in-silico* XFEM results were in good agreement with the *in-vitro* experimental observations. Field-emission scanning electron micrograph (FE-SEM) images (Fig. 5d–f) further revealed that the cracks initiated at the joints in the interface of glass phase and submicron crystals (Fig. 5f) and grew across the strut before branching to the neighbor struts. Figure 5f shows one primary crack initiation where a micro-crack initiated from the stress field caused by the difference in stiffness of submicron crystal phase from those of the other two constitutive phases.

**Figure 6:**
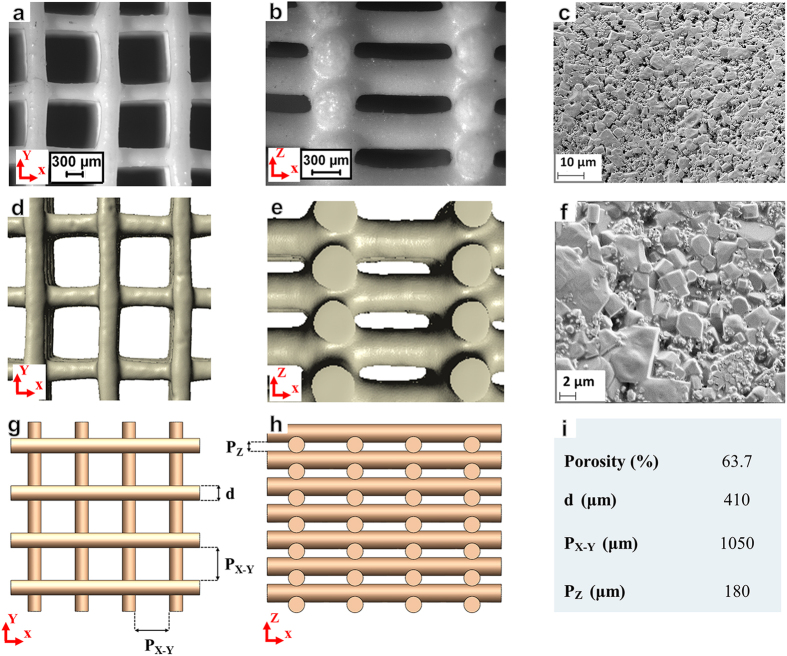
(**a**,**b**) Top and side views of the actual Sr-HT-Gahnite scaffolds fabricated in this study; (**c**,**f**) pore morphology and microstructure of the scaffolds; (**d**,**e**) top and side view of μCT-based models of actual fabricated scaffolds; (**g**,**h**) top and side views of post CAD models recreated based on average measurements from the actual fabricated scaffolds; (i) detailed geometrical features of the post CAD models.

While this study demonstrated the effectiveness of XFEM technique for predicting fracture behaviors of Sr-HT-Gahnite robocast scaffolds, some limitations exist. First, in order to improve the reliability of experimental data, more scaffold samples could be fabricated and tested experimentally. Second, the fabricated Sr-HT-Gahnite scaffolds contained micropores (Fig. 6c,f) and small defects such as air pockets (Fig. 5d) which were not considered in the numerical simulations. According to the Ashby model^28^, total volume fraction of pores in scaffolds known as porosity, can significantly influence their mechanical properties. However, the contribution of micropores to the total porosity of the scaffold structure could be negligible because the printed scaffolds mostly contained macropores with a size between 300 μm and 1000 μm that is around 20–100 times bigger than the micropore size. Additionally, Sr-HT-Gahnite offers an excellent sinterability due to liquid phase sintering mechanism for achieving fairly solid struts^29^. Therefore, the dominant factor in determining mechanical properties of printed scaffolds is macropores. Nevertheless, modeling the microstructure of the ceramic material^30^ and considering the effect of micropores on the fracture behaviors of the scaffolds remain a limitation of this study, which needs to be investigated in the future. Third, while reconstruction parameters were kept constant for each model to provide consistent greyscale intensity for the image based modeling analysis, the possible effect of different image processing parameters on the final modeling results was not investigated. Fourth, the possibility of introducing surface cracks in sawing and grinding the scaffolds was not considered in the *in-silico* simulations, which might change the crack initiation and propagation. Fifth, as fatigue may contribute to the failure of tissue scaffolds *in vivo*, the XFEM based fatigue crack growth should be considered in the future studies. Finally, XFEM provides a new analysis engine for design optimization of a scaffold structure to maximize its fracture resistance^31^.

In conclusion, ultimate strengths of ceramic scaffolds play a critical role in their functionality, especially when presented in load bearing applications. As a result, optimization of scaffold architecture through insightful analysis and modeling of fracture processes is critical to their applications in regenerating large bone defects under loads. Due to the inherent brittleness of ceramic materials, the modeling of crack initiation and propagation in the scaffolds becomes a key issue to understand the fracture behaviors realistically. The stress analyses based on conventional FEM are not able to model cracking process under loading, compromising their capability and reliability for fracture analyses of ceramic structures. A relatively new numerical method, namely XFEM, capable of modeling time-dependent cracking process was used in this study to simulate fracture in the robocast Sr-HT-Gahnite scaffolds. Different from the conventional finite element solutions reported in the literature, this technique enables the modeling of the resistance of the scaffold to fracture propagation in line with energy release rate within the material. The results proved that the XFEM solution is significantly more realistic for predicting fracture strength of the scaffolds structures compared with conventional FEM counterpart. Moreover, the capability of XFEM to predict the correct fracture path in ceramic scaffolds was demonstrated through correlating with the field-emission scanning electron micrograph analysis herein. This study concluded that XFEM can be used as an effective and reliable tool to model the fracture behaviors of ceramic scaffolds, thereby providing a robust framework for further optimization of load bearing tissue scaffolds *in silico*.

## Methods

The scaffolds fabricated in the current study were made of a newly developed ceramic material called Sr-HT-Gahnite. Details of the Sr-HT-Gahnite material preparation have been reported before^10^,^24^. The process related to the fabrication of Sr-HT-Gahnite scaffolds by direct ink writing (namely robocasting) technique, as well as characterization and experimental tests performed on scaffolds are briefly described below.

### Direct ink writing (robocasting) of Sr-HT-Gahnite scaffolds

Sr-HT-Gahnite scaffolds were fabricated by depositing the formulated in-house inks^24^ through a customized nozzle using a robotic deposition device (Hyrel 3D, USA). The prepared Sr-HT-Gahnite ink was first loaded into a syringe and then mounted on the robotic arm. The ink was deposited on an oil coated glass substrate (4 mm thickness). The printed scaffolds were easily detached from substrate after air-drying for 24 hours. A controlled-heat treatment was performed to decompose the organics and for sintering the particles into dense rods. The green samples were heated at a rate of 1 °C/min up to 450 °C and then densified at 1250 °C for 3 h. Prior to characterization, surface grinding was performed on the specimens to remove the solid walls and ensure that scaffold faces to be tested were flat and parallel. Figure 6a,b depicts the top (X-Y) and side (X-Z) views of the scaffold specimens fabricated in this study.

The scaffold specimens (n = 5) fabricated in the present study were composed of orthogonal layers of Sr-HT-Gahnite rods with a rectangular configuration having an average rods’ diameter of 410 μm. The geometric features related to these scaffolds that had dimensions of 3 mm × 3 mm × 3 mm are displayed in Fig. 6g–i. To further verify the numerical results obtained, our previous experimental results^24^ were also used here, where four different sets (n = 30) of scaffolds (6mm × 6mm × 6mm) were fabricated in the same structural configuration^24^. Note that these previously fabricated scaffolds had a different porosity and average rods’ diameter of 550 μm. The detailed geometric features related to these previous scaffolds^24^ are summarized in Fig. 1.

### Characterization and experimental tests

The scaffold specimens were first subjected to surface grinding to eliminate edge-effects and to obtain parallel testable surfaces. The free surface of scaffolds was further processed using a polishing pad to minimize any visible micro-cracks/defects on the surface. Field emission scanning electron microscopy, FE-SEM, (Zeiss Ultra plus, Germany) was used to observe the microstructure of the scaffolds. For this purpose, the scaffold specimens were sputter-coated with gold and then examined at an accelerating voltage of 5 kV. Figure 6c,f show pore morphology and microstructure of the scaffolds. The average porosity of the sintered scaffolds was measured using Micro-Computed Tomography (μCT) (SkyScan 1172).

The compressive strength of the scaffolds was tested in the direction parallel (clinically relevant position for the *in-vivo* defects) to the pore channels at a cross-head speed of 0.5 mm/min. The experiments were stopped when the scaffolds underwent catastrophic failure.

### Post CAD-based models

The post CAD model of each scaffold was created in SolidWorks and then imported into ABAQUS 6.13 (SIMULIA, Providence, RI, USA) for numerical analyses. It should be noted that the scaffold specimens undergo significant shrinkage during the fabrication process especially after sintering. Therefore, the post CAD models were created based on the average measurements of final post-fabricated scaffolds in order to take into account their dimensional shrinkage. In ABAQUS, the post CAD models were placed in between two parallel rigid plates; one of the rigid plates was fixed while the other could move vertically under a linearly ramped load. The force was applied perpendicularly to the scaffold plane (X-Y plane), simulating a compression test in the Z direction. It should be noted that a mesh convergence study was conducted to ensure a proper size of the elements for the numerical analysis. The geometric features related to the post CAD model of fabricated scaffolds are shown in Fig. 6g–i.

### μCT-based models

**Figure 7:**
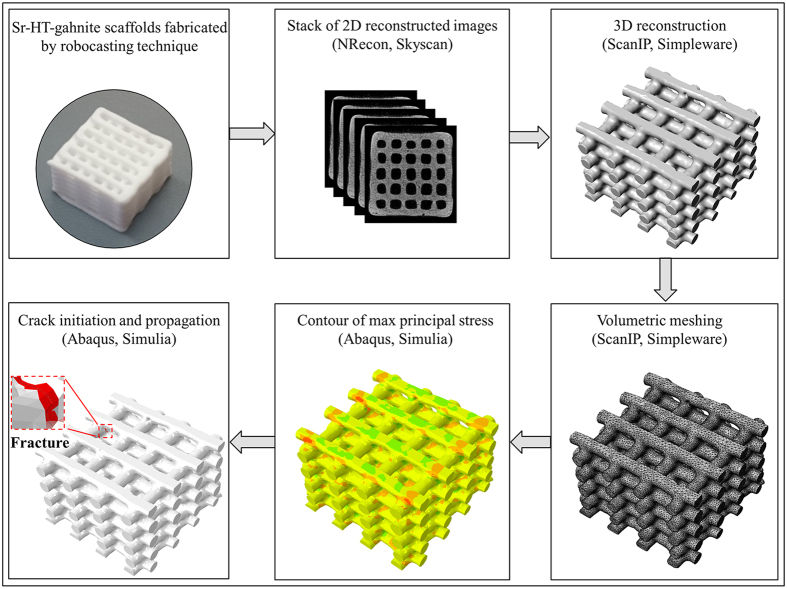
The flowchart demonstrates how the μCT-based XFEM analysis is performed on the fabricated Sr-HT-Gahnite scaffolds in silico.

To capture both dimensional and shape variations due to fabrication, the μCT-based models were created based on the μCT images from the actually fabricated scaffold specimens (n = 5). These five scaffolds have an average rods’ diameter of 410 μm which were particularly fabricated for fracture modeling analysis in this study. Figure 7 outlines the procedure for creating the μCT-based numerical models of the post-fabricated scaffolds. Each scaffold was scanned using SkyScan 1172 (Kontich, Belgium) at an 11 *μm* voxel size resolution with 100 *kV*, 100 *μA*, and a 1.0 *mm* aluminum filter. Projection images were integrated for 885 ms every 0.5° over a full 180° rotation. Each raw data set was then reconstructed into an axial stack saved as greyscale BMP images using SkyScan’s reconstruction software NRecon (Kontich, Belgium) for further processing. Reconstruction parameters were kept constant for each scan of specimen to provide consistent greyscale factors for the analysis.

Each set of reconstructed images was then imported into the image-processing software ScanIP (Simpleware Ltd, Exeter, UK) where a Recursive Gaussian filter with 18 *μm* was applied as a typical smoothing filter. The material was assumed to be homogeneous and mesh was then generated in four-node linear tetrahedral elements based on the +FE Free algorithm in ScanFE (Simpleware Ltd, Exeter, UK). Each μCT-based model was imported into ABAQUS 6.13 where the boundary and loading conditions were applied on each model as same as the CAD-based models. Figure 6d,e depicts the top and side views of μCT-based models of the actual fabricated scaffolds.

### Material properties

In this study, it was assumed that the scaffold material was homogeneous and isotropic after the fabrication. The flexural strength (97 ± 11 MPa) was measured by conducting a three-point-bending test (ASTM C1684) using the rectangular bars (4 mm × 5 mm × 35 mm) with a span length of 25 mm and a crosshead loading speed of 0.5 mm/min in the center. Other relevant material properties were sourced from the literature^10^,^32^,^33^. The Young’s modulus of Sr-HT-Gahnite is 40 ± 7 GPa, the Poisson’s ratio is 0.26, and the critical fracture toughness is 1.24 Pa.m^1/2^.

### Brief of XFEM

In this study, XFEM was employed through commercial code ABAQUS 6.13 (SIMULIA, Providence, RI, USA) to simulate crack initiation and propagation in the Sr-HT-Gahnite scaffolds. XFEM enables simulating crack onset and growth under the assumptions of linear elastic fracture mechanics (LEFM)^34^,^35^. This technique needs to define a part or an area of a part as an enriched zone where a crack can initiate and grow. The XFEM enriched environment can be used to look closely at fracture failure by capturing the jump of field variables in the cracked domain; therefore the description for the discontinuous field was independent of the mesh^36^.

Specifically in this study, the maximum principal stress-based damage criterion was adopted. The displacement field variable *u*^*h*^ is enriched by the additional functions using the partition of unity framework, as follows,


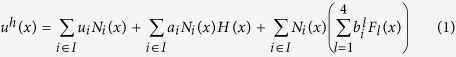


where *I* is the set of nodes in the mesh, 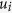 is the normal degree of freedom at node *i*. 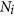 is the standard shape function associated with node *i*. 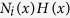 is the enriched shape function. 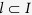 is the subset of nodes enriched by the Heaviside function 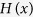,


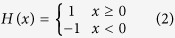


Function 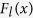, *l* = 1, …, 4 is used for modeling the full radial dependence crack tip, given as,


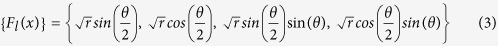


where (*r*, *θ*) is a polar coordinate system with its origin at the crack tip and *θ* = 0 is tangent to the crack at the tip.

Crack initiation was determined in terms of the ratio of the maximum tensile stress 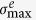 to allowable stress 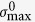 in an element, as,


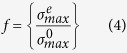


Onset of damage takes place when the stress ratio equals one (*f* = 1) as indicated in equation (4).

In this study, an effective separation was defined to describe the evolution of damage under a combination of normal and shear separation across the interface,


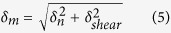


where *δ*_*m*_ is the total mixed-mode relative displacement, *δ*_*n*_ the normal relative displacement, *δ*_*shear*_ the tangential displacements of the element.

Crack propagation was determined based upon energy release rate within the material. Following a crack initiation, the propagation follows as per whether the strain energy release rate *G* of the concerned materials can be achieved, which is derived from the Irwin fracture condition^26^, as


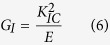


where *K*_*IC*_ is the stress intensity factor in mode I fracture. The mixed mode of power law criterion was used for quantifying the damage evolution as expressed in equation (7).


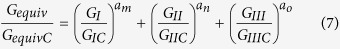


where 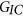 is energy release rate in Mode I, 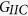 in Mode II, and 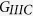 in Mode III, 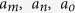 are power exponents and are set equal to 1 in this study^22^,^37^,^38^. In the case of a homogenous stress field, the crack propagates in the direction perpendicular to the maximum tensile stress.

### Statistics

All the experimental data and μCT-based numerical results related to the compressive strength of Sr-HT-Gahnite scaffolds fabricated in the current study have been presented as mean ± SD (Fig. 3), and were derived from the five independent samples. For the statistical analyses, ANOVA followed by Tukey’s multiple comparisons test was used. The PASW statistics program was employed for all the statistical analyses and the differences were considered as significant if p < 0.05.

## Additional Information

**How to cite this article**: Entezari, A. *et al*. Fracture behaviors of ceramic tissue scaffolds for load bearing applications. *Sci. Rep.* **6**, 28816; doi: 10.1038/srep28816 (2016).
